# Difficulties in Emotion Regulation, Alexithymia, and Social Phobia Are Associated With Disordered Eating in Male and Female Undergraduate Athletes

**DOI:** 10.3389/fpsyg.2020.01646

**Published:** 2020-07-22

**Authors:** Erik M. Benau, Ryan Wiatrowski, C. Alix Timko

**Affiliations:** ^1^Department of Psychology, University of Kansas, Lawrence, KS, United States; ^2^Department of Psychology, Towson University, Towson, MD, United States; ^3^Department of Child and Adolescent Psychiatry and Behavioral Sciences, Children’s Hospital of Philadelphia, Philadelphia, PA, United States

**Keywords:** social cognition, drive for thinness, binge eating, body dissatisfaction, emotion recognition

## Abstract

Investigations of disordered eating in the athlete population tend to focus on females and the influence of sport level. This leaves unanswered whether, and how, team interdependence (i.e., whether the competition is engaged with one person or as a team) may differentially impact male athletes. In the present study, we recruited a sample of non-athletes, individual athletes, and team athletes and examined the interaction of gender and teammate interdependence on established psychosocial risk factors for disordered eating, including social phobia, alexithymia, and emotion regulation. Although we identified a significant main effect of gender, there was no main effect of team type, nor was there a significant interaction of gender and team type. Using descriptive discriminant analysis, these variables significantly discriminated between genders. Women were defined by higher scores than men on drive for thinness, body dissatisfaction, and emotion recognition and men were defined by relatively higher scores on emotion dysregulation and binge eating. When we combined all athletes and compared them with non-athletes, a significant interaction of gender and athlete status emerged such that female athletes, compared to male athletes and women non-athletes, were defined by higher scores on drive for thinness, emotion dysregulation, and binge eating. Conversely, male athletes, compared to female athletes, were defined by greater difficulty identifying feelings and body dissatisfaction. Non-athletes were not well defined by the discriminant function. These results highlight that emotional processes convey risk of eating disorders in men and women, particularly in athletes, and these risk factors are not uniform.

## Introduction

A large volume of work spanning nearly three decades has identified the Female Athlete Triad, a syndrome consisting of disordered eating, amenorrhea, and osteoporosis (Yeager et al., 1993; Otis et al., 1997; Ranson et al., 2018). Although these three signs tend to be the most commonly thought of, the triad syndrome has been updated to include broader conditions: low energy availability (with or without disordered eating), menstrual dysfunction, and low bone mineral density (Nattiv et al., 2007; Williams et al., 2017). Recently, a parallel to the Female Athlete Triad has been proposed for male athletes that comprises hypogonadotropic hypogonadism, low energy availability, and low mineral bone density (Tenforde et al., 2016). The effects of the male triad appear to be less frequent, less severe, and more treatable than the female triad (De Souza et al., 2019). Central to the athlete triad of both men and women is low energy availability, which is frequently exacerbated by disordered eating (Williams et al., 2017). For both men and women, the etiology of the triad is similar: pressure to enhance performance or maintain an advantageous aesthetic can lead to an athlete expending excessive energy in their exercise and consuming insufficient nutrients to maintain homeostasis (Torstveit et al., 2008; Sundgot-Borgen and Torstveit, 2010; Ranson et al., 2018; De Souza et al., 2019).

Investigations of disordered eating in athletes have tended to focus on assessing prevalence and risk across sports and sport levels, particularly for women, and as a result, the psychosocial mechanisms of risk for athletes compared to non-athletes are not well understood (Papathomas and Lavallee, 2012; Papathomas and Petrie, 2014). The results of these studies typically suggest that female athletes are at increased risk of disordered eating compared to male athletes and female non-athletes (Varnes et al., 2013; Joy et al., 2016); this is particularly true for female athletes in lean or aesthetically influenced sports (e.g., ice skating, gymnastics).

Although published studies including male athletes are less common, men and boys may be at greater risk of disordered eating than women and girls when the type of sport, social environment of teams, assessment used, and specific disordered eating behaviors are examined (Baum, 2006; Papathomas and Lavallee, 2012; Papathomas and Petrie, 2014; Pope et al., 2015; Chapman and Woodman, 2016; Joy et al., 2016; Rice et al., 2016). Moreover, the social environment and teammate interdependence (i.e., whether the competition is completed alone or as a team) can play a key role in physical and psychological outcomes of male and female athletes (Evans et al., 2012; Martin et al., 2014), including in eating disorder risk (Scott et al., 2019a, b). Given the influence of peer groups in the risk of eating disorders (Keel and Forney, 2013), team dynamics may be an important aspect of disordered eating risk that should be investigated further.

Closer examination of the social environment of teams, particularly team interdependence, indicates important distinctions between male and female athletes’ disordered eating risk (e.g., Baum, 2006; Galli and Reel, 2009). Some have found girls in team sports reported greater body dissatisfaction and eating disorder symptoms than girls in individual sports or their male counterparts in either sport type (Dyremyhr et al., 2014), although others have found the opposite (Haase, 2009). Although female athletes engaged in team sports have reported many positive aspects of their experience, they also have reported increased body-related social comparison and perceived scrutiny of their weight and appearance from teammates, coaches, and family (Crissey and Honea, 2006; Frisen and Holmqvist, 2010; Kosteli et al., 2014). Conversely, for boys and men, team sports have been found to offer benefits that can protect against eating disorder onset, such as social facilitation, encouragement, and camaraderie (Baum, 2006; Galli and Reel, 2009; Frisen and Holmqvist, 2010; Morano et al., 2011; Chapman and Woodman, 2016). Men and boys in individual sports (particularly those that are gravitational and weight class–based) have reported disordered eating at rates and severity greater than males in other sports and comparable to, or exceeding, female athletes (Morano et al., 2011; Joy et al., 2016).

There is also a notable gap in the literature pertaining to undergraduate athletes who engage in sports at the club or recreational level (Bratland-Sanda and Sundgot-Borgen, 2013; Martinsen and Sundgot-Borgen, 2013; Manore, 2015). Previous data provide consistent evidence that women elite athletes (i.e., collegiate, “Division 1,” professional, etc.) are at increased risk of disordered eating than non-athletes (Martinsen and Sundgot-Borgen, 2013; Cosh et al., 2014; Rice et al., 2016; De Bruin and Oudejans, 2018). To our knowledge, only three published studies have examined eating disorder risk in samples of recreational and club athletes. Two of these studies featured exclusively female participants (Holm-Denoma et al., 2009; Swami et al., 2009) and found that women involved in recreational sports were at increased risk of disordered eating than non-athletes but were at reduced risk compared to elite athletes. An additional study examined the interaction of gender and competition level (Palermo and Rancourt, 2019); the results showed a positive relation between competitive level of sport and disordered eating risk, which did not significantly differ between men and women. Palermo and Rancourt suggest that recreational undergraduate athletes may be overrepresented by individuals who were competitive athletes in high school and thus carried over some of the “athlete identity” and eating disorder risk present prior to matriculating to college.

Compared to research into competition level, sport type, and desire/pressure for performance enhancement as risk factors for disordered eating, there is notably less research probing psychosocial and socioemotional variables in the athlete population (Papathomas and Lavallee, 2012; Papathomas and Petrie, 2014). It is critical to address this gap in order to understand the specific needs of athletes in the treatment and prevention of disordered eating (De Bruin, 2017). Although some well-established risk factors for disordered have been confirmed in college athletes, including perfectionism (Forsberg and Lock, 2006), “athletic identity” (Palermo and Rancourt, 2019), and social desirability (Petrie et al., 2007; Greenleaf et al., 2010), much more research is needed. Two main constructs related to disordered eating in both men and women in the general population have surprisingly limited or no examinations within the athlete population: emotion regulation (Lavender and Anderson, 2010; Griffiths et al., 2014; Lavender et al., 2015; Wyssen et al., 2016; Haynos et al., 2018) and social cognition (Kerr-Gaffney et al., 2018; Leppanen et al., 2018).

Emotion regulation refers to an ability to accurately identify and modify one’s emotional experience (Aldao et al., 2010; Gratz and Roemer, 2004). Emotion regulation is underpinned by *emotional literacy* or the ability to identify emotions of self and others; when this process is impaired, it is referred to as *alexithymia* (Kooiman et al., 2002; Taylor et al., 2003). Alexithymia has been consistently associated with disordered eating, wherein either overeating or restrictive eating can serve as a coping mechanism for emotional distress (Aldao et al., 2010; Nowakowski et al., 2013; Westwood et al., 2017). Individuals who exhibit emotional dysregulation and/or alexithymia also tend to demonstrate impaired recognition of representations of emotions in others, such as in pictures (Kessler et al., 2006; Lyvers et al., 2017). Poor performance in emotion recognition tasks has been associated with disordered eating (Medina-Pradas et al., 2012; Leppanen et al., 2018). A few studies indicate that athletes exhibit a similar relation between emotion dysregulation and disordered eating, yet most of these studies investigated this relation with self-report measures in exclusively female samples and did not examine sport type (e.g., Wollenberg et al., 2015; Shriver et al., 2016). Thus, it is not yet established whether deficits in emotion regulation, including alexithymia and emotion recognition, are equally risky for male athletes and/or athletes with different social environments in their sport.

Social cognition is a broad set of skills that contribute to the ability to process social information, particularly the thoughts and feelings of others (Gkika et al., 2018). Like alexithymia, social cognition is centered on recognizing the emotions in others, including identifying emotions portrayed in pictures as described above (O’Toole et al., 2013; Gkika et al., 2018). Deficits in social and emotional cognition also are linked social phobia (sometimes referred to as social anxiety disorder), which is a fear of negative evaluations from others (O’Toole et al., 2013; Gkika et al., 2018) and eating pathology (Kerr-Gaffney et al., 2018; Leppanen et al., 2018). There is evidence that performance on social cognition tasks and prevalence of social phobia are consistently higher in women than men (Sutterby et al., 2012; Kirkland et al., 2013; Vellante et al., 2013). This association suggests that women, compared to men, are more sensitive to the evaluations of others and in turn contributes to their comparatively higher rates of disordered eating (Kerr-Gaffney et al., 2018, 2019). Given the high association of disordered eating with social phobia and social cognition, it is surprising that, to our knowledge, no study has investigated these associations specifically in college athletes.

Positive outcomes in athletics are predicated on appropriate, competent management of intrapersonal (emotional) and interpersonal (social) demands and processes regardless of sport type (Martin et al., 2014; Eys et al., 2015). Therefore, social cognition, including social phobia, and emotion regulation are intuitive and important avenues to pursue as a potential risk and protective factors across sports for many outcomes, including disordered eating. Establishing whether, and how, these relations are present in athletes can provide a target for further research and, ultimately, tailored treatment and prevention programs for disordered eating in this population.

## Current Study

The aim of the current study was to synthesize and extend findings from recent literature on psychosocial risk for disordered eating in undergraduates and in athletes. We sought to determine whether male or female athletes had greater difficulty in domains of social cognition, emotion regulation, and emotion recognition. By examining the impact of teammate interdependence and gender on disordered eating and social cognition, we hope to build a model for the role of social cognition as either a risk or maintenance factor for disordered eating in athletes that can be tested in future prospective studies.

To address these aims, we recruited a sample of undergraduate athletes and non-athletes who completed a series of instruments that assess alexithymia, emotion regulation, social phobia, emotion recognition, and eating disorder symptomatology. Non-athletes were included as a control group within the sample population. We hypothesized that difficulties in social cognition would be correlated with greater disordered eating risk of athletes and non-athletes. Further, the socially interdependent nature of team sports would be associated with stronger emotion recognition and regulation skills than individual or non-athletes. We hypothesized that female athletes involved in team sports would have greater eating disorder risk than those in individual sports or men of any sport. We also hypothesized the inverse to be true for men: those on individual sports would show greater eating disorder risk than those on teams.

## Materials and Methods

### Participants

A total of 293 self-selected undergraduates at a large Mid-Atlantic university completed the survey in exchange for course credit. Thirteen participants (4%) were removed for missing data (we did not impute or replace any missing responses) and one whose age (45 years) was an outlier, leaving a final sample of 279 respondents (177 women where gender was inquired as a binary choice). The median age of respondents was 19.0 years (mean = 19.3 years, *SD* = 2.24 years, range = 18–26 years). Most were 21 years or younger (90%; *n* = 250). The race and ethnic composition of the sample broadly matched the composition of the university: 79% were White, 12% African American, 3% Hispanic, 2% Asian/Pacific Islander, < 1% Native American/Native Alaskan, and 2% other.

### Measures

#### Eating Disorders Inventory, Third Edition (EDI-3; Garner, 2002)

The 91-item EDI-3 is a reliable and valid instrument that evaluates a range of behavioral and psychological symptoms pertaining to risk of onset or maintenance of disordered eating (e.g., Stanford and Lemberg, 2012). Questions are on a six-point Likert-type scale ranging from 1 (*not at all*) to 6 (*extremely*). Previous research using the EDI-3 within the athlete populations found that it maintains its validity in those samples, with more available data for women than men (Pope et al., 2015; Chapman and Woodman, 2016). We scored the instrument in accordance with the manual whereby scores are truncated to a scale of 1 to 4 referring to symptomatic (moderate–extreme) levels of a stated thought or behavior (Garner, 2002).

For the purposes of the present study, we analyzed the three subscales most pertinent to eating disorder risk: drive for thinness (EDI-DT; seven items), body dissatisfaction (EDI-BD; 10 items), and bulimia (EDI-B; eight items). The EDI-DT scale examines anxiety about gaining weight, and the EDI-BD evaluates perceived unhappiness with the perceived shape of one’s body, particularly the hips, buttocks, and legs. As others have noted (e.g., Stanford and Lemberg, 2012), the EDI-B scale evaluates binging more so than the purging behavior associated with bulimia. The sample exhibited good internal consistency on the three subscales (all α > 0.80).

#### Toronto Alexithymia Scale-20 (TAS-20; Bagby et al., 1986)

The TAS-20 is a validated and reliable 20-item scale of alexithymia (Bagby et al., 1994a, b; Parker et al., 2003) and has been previously used to assess psychosocial risk in the student athlete population (e.g., Cazenave et al., 2007). The TAS-20 has three subscales: difficulty identifying feelings (TAS-DIF; seven items), difficulty describing feelings (TAS-DDF; five items), and externally oriented thinking (TAS-EOT; eight items). The TAS-DDF assesses impairment in properly labeling an emotion; the TAS-DIF probes impaired ability to describe an emotional experience and the context of emotions, and the TAS-EOT measures a belief that emotions are controlled by external stimuli. Items are rated on a scale of 1 (*strongly disagree*) to 5 (*strongly agree*). The TAS-DDF and TAS-DIF subscales are consistently associated with disordered eating, although the TAS-EOT subscale is not (Nowakowski et al., 2013; Westwood et al., 2017). Thus, we excluded the TAS-EOT from analyses. The sample exhibited good internal consistence on the TAS-DDF and TAS-DIF (α > 0.79).

#### Difficulties in Emotion Regulation Scale (DERS; Gratz and Roemer, 2004)

The 36-item DERS measures the emotional regulation and subjective emotional experience (Gratz and Roemer, 2004). Items are reported on a scale of 1 (*almost never*) to 5 (*almost always*), with higher scores corresponding to greater difficulties (Gratz and Roemer, 2004). The scale is valid and reliable, unbiased based on gender, and predictive of clinical and non-clinical disordered eating in collegiate athlete populations (Lavender and Anderson, 2010; Lavender et al., 2015; Ritschel et al., 2015; Wollenberg et al., 2015; Shriver et al., 2016; Hallion et al., 2018; Haynos et al., 2018). We analyzed the sum score of the instrument. The present sample demonstrated good internal consistency on the scale (α = 0.82).

#### Social Phobia Inventory (SPIN; Connor et al., 2000)

The 17-item SPIN probes symptoms of social phobia on a scale of 0 (*not at all*) to 4 (*extremely*). It is valid and reliable in clinical and non-clinical samples (e.g., Connor et al., 2000; Radomsky et al., 2006). We included this measure as social phobia can occur when social cognition is impaired, typically due to heightened concerns about the judgments of others (Sutterby et al., 2012; O’Toole et al., 2013) that can, in turn, increase the risk of disordered eating (Keel and Forney, 2013; Kerr-Gaffney et al., 2018). We used the sum score of the instrument in the present study. The sample demonstrated excellent internal consistency on the scale (α = 0.92).

#### Reading the Mind in the Eyes Task–Revised (RMET; Baron-Cohen et al., 2001)

The RMET examines a component of Theory of Mind related to the “attribution of the relevant mental state” (Baron-Cohen et al., 2001, p. 241). Participants are presented a series of 36 gray-scale photographs of different individuals (counterbalanced for gender), which are cropped to the eye area of the face. Participants choose one of four emotions that best describe what the person in the photograph is feeling. We included the RMET as a measure of the recognition of emotions in others, a key component in emotion regulation (Oakley et al., 2016). Performance on the task is negatively associated with disordered eating (Bora and Kose, 2016; Kerr-Gaffney et al., 2019). The internal consistency of the RMET is best achieved using confirmatory factor analysis (Vellante et al., 2013). The present sample had strong goodness of fit to a unitary model, χ^2^(594) = 748.63, *p* < 0.001, root mean square error of approximation = 0.03, *pclose* = 1.0; standardized root mean residual = 0.060.

#### Athletic Participation

We asked participants to list all organized sports in which they were currently (or most recently) engaged, starting with present participation. They were instructed to not include informal or self-initiated exercise (e.g., personal exercise routines). They also identified whether the competition of their sport was engaged as a team or individually. We changed 10 individuals’ selected classifications of team and individual sports to be consistent with the recommendations of Evans et al. (2012) based on level of structural teammate interdependence (whether/how teammates are involved in the actual competition of the sport). Maintaining the original self-reported teammate interdependence level did not change significance in the analyses described below. They indicated level of participation (club, recreational,^1^ collegiate, professional, or “other” with the option to fill in) and whether the sport was in season.

### Procedure

The institutional review board approved all procedures. Participants completed informed consent and questionnaire confidentially at scheduled times in a group setting in a laboratory on campus. We used online survey software (SurveyMonkey; SurveyMonkey, Inc., Palo Alto, CA, United States). After completion of the study, participants received debriefing information.

#### Data Analysis

We conducted a series of χ^2^ analyses to evaluate the distribution of gender and sport type and a Mann-Whitney *U*-test to assess number of sports by gender. We conducted Pearson correlations between each of the assessments, age, body mass index (BMI), and gender to establish whether each of these items was independently associated with each other. We conducted Kendall τ_*b*_ between these variables and sport type. We conducted a 2 (gender: men, women) × 3 (team interdependence: non-athlete, team, individual) multivariate analysis of covariance (MANCOVA) with the scores of the eight variables discussed above as dependent variables (EDI-DT, EDI-BD, EDI-B, TAS-DIF, and TAS-DDF subscales, and the sum of the DERS, SPIN, and RMET) and BMI entered as a covariate. In lieu of follow-up univariate tests and pairwise comparisons, we conducted descriptive discriminant analyses (DDAs), based on the recommendations of Barton et al. (2016). Descriptive discriminant analysis has the advantage of reducing type 1 error and is suited to differentiate groups based on which variables best describe them dimensionally and in combination (Sherry, 2006).

Descriptive discriminant analysis is mathematically and conceptually similar to multiple regression and factor analysis. The variables are factor analyzed, generating a synthetic composite akin to a factor. The results provide composite scores (i.e., a structure *r*; *r*_*s*_) that describe the strength and direction (positive or negative) of each variable’s association with the composite. Group differences are assessed via group centroids, which are the average (mean) degree that this composite defines the groups. Both *r*_*s*_ and group centroids range from −1 to + 1. These describe the strength of association of the group to the composite and its variables. There is no broadly accepted benchmark for whether the absolute value of a *r*_*s*_ or centroid is meaningful (Huberty and Olejnik, 2006; Sherry, 2006; Barton et al., 2016). For the purposes of the present study, we used a cutoff of |0.17| for *r*_*s*_ and group centroids as our power analyses support correlations this size (Finch and Laking, 2008). Correlations at this level with the present sample size are unlikely to be an error (Murphy et al., 2014). Larger absolute values of group centroids indicate greater definition by the factor, whereas centroids closer to zero indicate poorer definition by the factor. Negative centroids indicate that the direction of association for the group in the synthetic composite is reversed. We controlled for BMI in the DDA by regressing each dependent variable onto BMI and calculating the standardized residuals for each variable. These residuals served as the variables entered into the DDA model.

Several of the variables in the present study violated assumptions of normality (Shapiro–Wilks and Kolmogorov–Smirnov *p*’s < 0.01), which is not surprising given the relatively large size of the present sample and that several variables are one-tailed by design (e.g., social phobia symptoms) (Blanca et al., 2013; Delacre et al., 2019). Levene’s test indicated the homogeneity of variance assumption was met for each variable when comparing gender (*p*’s > 0.07). The variance was significant when comparing team type for the DERS and TAS-DIF (*p*’s < 0.01). However, *F*-tests (i.e., the MANCOVA) and the DDA we utilized are robust to even severe violations of these assumptions (Finch, 2009; Sherry, 2006; Blanca et al., 2017). Thus, we argue it is safe to cautiously proceed with these analyses. The remaining assumptions of multivariate analysis of variance (MANOVA) and DDA (e.g., random sampling, interval measurement, multicollinearity) were met (Sherry, 2006).^2^ Sensitivity analysis by G^∗^Power software (Faul et al., 2007; Faul et al., 2009) indicates that our sample was sufficient to power (1 - β ≥ 0.80) small to medium effect sizes for the MANCOVA and DDA, *f* = 0.19 (*R*^2^ = 0.16), correlations of *r* > 0.17, and pairwise comparisons with *d* > 0.35.

## Results

### Teammate Interdependence

Participants reported involvement in more than 21 organized sports as their first reported sport (Supplementary Appendix A). There was a range of athletic participation, with 183 participants (66%) reporting engagement in at least one sport and the remaining 96 (34%) reporting no sport engagement (i.e., non-athletes). Of the non-athletes, 28 (29%) were men and 68 (71%) were women. Of the athletes, 138 (75%) played a team sport (i.e., team athletes), of whom 62 (45%) were men and 76 (55%) were women. The remaining 45 athletes (25%) played individual sports (i.e., individual athletes), 13 (29%) of whom were men and 32 (71%) were women. Most sports (*n* = 116; 63%) were in season, which was comparably represented across team and individual sports, as well as genders (*p*’s > 0.07, *V*’s < 0.08).

Most athletes (64%) were at the recreational (*n* = 42; 23%) and club level (*n* = 74; 41%), with the rest at the collegiate (*n* = 63; 35%) and semiprofessional and professional levels (*n* = 2; 1%). Two (1%) did not provide their level of participation. None reported an “other” level. Across sport levels (e.g., club, collegiate), there was a comparable distribution of team and individual sports χ^2^(4) = 7.14, *p* = 0.129, *V* = 0.20, and gender χ^2^(4) = 4.36, *p* = 0.359, *V* = 0.16. Removing the four respondents who were professional or semiprofessional or who did not respond did not change the significance of these distributions (*p*’s > 0.12) and did not change significance, direction, or magnitude of the results described below.

Men were somewhat overrepresented as team athletes, χ^2^(2) = 7.52 *p* = 0.023, *V* = 0.164, but were comparably represented as individual and non-athletes (*p*’s > 0.05). The proportion of men and women was statistically comparable in non-athletes and individual athletes (*p*’s > 0.05). In terms of BMI, univariate analysis of variance indicated that there were no significant main effects or interactions of gender and team type (*p*’s > 0.097).

### Relation of Sport Type, Gender, and Psychosocial Variables

Descriptive statistics for questionnaire data are presented in Table 1. The correlations between each of the assessments, age, BMI, and gender, are shown in Table 2. Note that the SPIN positively correlated with most measures for both men and women. Worse performance on the RMET significantly correlated with both alexithymia subscales (TAS-DDF, TAS-DIF) and the EDI-B, but only for men. Body mass index significantly correlated with nearly all measures except the SPIN and RMET for women, whereas for men, BMI correlated only with EDI-DT and EDI-BD. Sport type was not meaningfully correlated with any measure (all τ_*b*_ < | 0.1|).

**TABLE 1:** Descriptive statistics for questionnaire data.

Measure	Sport type	Men (*n* = 102)	Women (*n* = 177)	Sample (*n* = 209)
EDI-DT	No sport	6.32 (6.78)	9.29 (6.93)	8.43 (6.99)
	Team	2.97 (3.05)	10.07 (7.78)	6.88 (7.06)
	Individual	3.69 (3.71)	10.72 (7.24)	8.69 (7.14)
	*All Athletes*	*3.33 (3.38)*	*10.39 (7.51)*	*7.78 (7.10)*
	**Sample**	**3.97 (4.64)**	**9.89 (7.34)**	**7.7 (7.07)**
EDI-BD	No sport	9.39 (8.23)	14.66 (9.47)	13.13 (9.4)
	Team	6.60 (4.6)	14.04 (10.08)	10.7 (8.88)
	Individual	5.62 (4.41)	12.84 (7.59)	10.76 (7.54)
	*All Athletes*	*6.11 (4.5)*	*13.44 (8.83)*	*10.73 (8.21)*
	**Sample**	**7.23 (5.89)**	**14.06 (9.41)**	**11.54 (8.91)**
EDI-B	No sport	4.14 (5.3)	3.44 (4.18)	3.65 (4.52)
	Team	2.55 (2.55)	4.07 (5.14)	3.38 (4.23)
	Individual	2.77 (2.65)	3.75 (4.19)	3.47 (3.81)
	*All Athletes*	*2.66 (2.6)*	*3.91 (4.66)*	*3.43 (4.02)*
	**Sample**	**3.01 (3.56)**	**3.77 (4.6)**	**3.49 (4.26)**
DERS	No sport	85.54 (23.87)	76.01 (18.4)	78.79 (20.49)
	Team	78.6 (22.39)	76.45 (24.67)	77.41 (23.61)
	Individual	71.46 (16.8)	69.88 (14.11)	70.33 (14.76)
	*All Athletes*	*75.03 (19.59)*	*73.16 (19.39)*	*73.87 (19.19)*
	**Sample**	**79.58 (22.42)**	**75.09 (20.78)**	**76.75 (21.47)**
TAS-DIF	No sport	13.39 (6.34)	13.04 (4.39)	13.15 (5.00)
	Team	14.06 (6.7)	12.82 (6.05)	13.38 (6.36)
	Individual	13.23 (5)	11.22 (4.64)	11.8 (4.78)
	*All Athletes*	*13.65 (5.85)*	*12.02 (5.35)*	*12.59 (5.57)*
	**Sample**	**13.78 (6.37)**	**12.61 (5.23)**	**13.04 (5.69)**
TAS-DDF	No sport	12.36 (4.09)	11.71 (4.23)	11.9 (4.18)
	Team	11.82 (4.11)	11.09 (4.45)	11.42 (4.3)
	Individual	10.62 (3.36)	10.97 (4.36)	10.87 (4.06)
	*All Athletes*	*11.22 (3.73)*	*11.03 (4.4)*	*11.14 (4.18)*
	**Sample**	**11.82 (4.01)**	**11.31 (4.33)**	**11.49 (4.22)**
SPIN	No sport	21.07 (15.5)	20.35 (11.41)	20.56 (12.66)
	Team	16.56 (10.56)	16.01 (12.08)	16.26 (11.39)
	Individual	16.15 (10.08)	18.66 (12.01)	17.93 (11.43)
	*All Athletes*	*16.36 (10.32)*	*17.33 (12.05)*	*17.1 (11.41)*
	**Sample**	**17.74 (12.1)**	**18.17 (11.91)**	**18.01 (11.96)**
RMET	No sport	25.43 (5.15)	27.44 (3.24)	26.85 (3.97)
	Team	25.11 (4.88)	27.04 (3.38)	26.17 (4.22)
	Individual	26.38 (3.71)	26.28 (3.48)	26.31 (3.51)
	*All Athletes*	*25.75 (4.29)*	*26.66 (3.43)*	*26.24 (3.86)*
	**Sample**	**25.36 (4.80)**	**27.06 (3.35)**	**26.43 (4.02)**

**Values are presented as [M (SD)] and are based on raw scores; EDI-DT, Eating Disorders Inventory [EDI] Drive for Thinness subscale; EDI-BD, EDI Body Dissatisfaction Subscale; EDI-B, EDI Bulimia Subscale; DERS, Difficulties in Emotion Regulation Scale (total score); TAS-DIF, Toronto Alexithymia Scale Difficulty Identifying Feelings subscale; TAS-DDF, Toronto Alexithymia Scale, Difficulty Describing Feelings subscale; SPIN, Social Phobia Inventory; RMET, Reading Mind in the Eyes Task; BMI, Body Mass Index;* Team Athletes: Men = 62, Women = 76; Individual Athletes: Men = 13, Women = 32; Non-athletes: Men = 40, Women = 68; All Athletes: Men = 87, Women = 96. Bold = Descriptive values for the whole sample; Italics = Descriptive values for all athletes combined (individual and team).*

**TABLE 2:** Pearson bivariate correlations between the variables of interest for men (below dashes; *n* = 102) and women (above dashes; *n* = 177).

	Age	BMI	EDI-DT	EDI-B	EDI-BD	DERS	TAS-DIF	TAS-DDF	SPIN	RMET
Age	−	0.219**	–0.080	–0.06	–0.103	–0.027	–0.095	–0.116	−0.132†	–0.090
BMI	–0.073	−	0.268***	0.213**	0.397***	0.236**	0.160*	0.148†	0.048	–0.069
EDI-DT	0.177†	0.358***	−	0.571***	0.781***	0.315***	0.271***	0.147†	0.273***	–0.021
EDI-B	0.108	0.112	0.601***	−	0.499***	0.350***	0.417***	0.284***	0.253**	–0.097
EDI-BD	0.090	0.340***	0.669***	0.516***	−	0.406***	0.381***	0.270***	0.321***	–0.043
DERS	0.125	–0.058	0.280**	0.351***	0.413***	−	0.654***	0.462***	0.447***	–0.093
TAS-DIF	0.052	–0.043	0.191	0.261**	0.307**	0.741***	−	0.570***	0.421***	–0.074
TAS-DDF	0.128	–0.112	0.087	0.160	0.262**	0.650***	0.696***	−	0.329***	–0.137
SPIN	0.299**	–0.026	0.358***	0.359***	0.299**	0.435***	0.381***	0.470***	−	–0.032
RMET	0.017	0.019	–0.179	−0.211*	–0.090	−0.247*	−0.271**	–0.106	–0.087	−

BMI, Body Mass Index; EDI-DT, Eating Disorders Inventory-Drive for Thinness; EDI-B, EDI Bulimia scale; EDI-BD, EDI Body Dissatisfaction scale; DERS, Difficulties in Emotion Regulation Scale (total score); TAS-DIF, Toronto Alexithymia Scale Difficulty Identifying Feelings subscale; TAS-DID, Toronto Alexithymia Scale, Difficulty Describing Feelings subscale; SPIN, Social Phobia Inventory; RMET, Reading Mind in the Eyes Task; EDI, Eating Disorders Inventory; all values are rounded. ^†^p < 0.10; * p < 0.05; ** p < 0.01; *** p < 0.001.

The omnibus MANCOVA showed a significant main effect of gender, *F*(8, 266) = 8.16, *p* < 0.001, η*_*p*_*^2^ = 0.197. However, the main effect of team type, *F*(16, 532) = 1.16, *p* = 0.297, η*_*p*_*^2^ = 0.034, and the team type × gender interaction were not significant, *F*(16, 532) = 1.30, *p* = 0.189, η*_*p*_*^2^ = 0.038. Because only the main effect of gender was significant, we performed the DDA accordingly. Group centroids and both standardized and raw *r*_*s*_ for each variable in this function are found in Table 3. Gender explained approximately 30% of the variance in the composite, Wilks’ and Levene’s and Welch’s tests are possessive Λ = 0.702, χ^2^(8) = 94.85, *p* < 0.001, *R*_*c*_^2^ = 0.298. As shown in the standardized canonical coefficients, the function was positively defined by EDI-DT, EDI-BD, and RMET. The function was negatively defined by DERS and EDI-B. TAS-DIF, TAS-DDF, and SPIN did not meaningfully contribute to the variance of the function (standardized *r*_*s*_ < 0.17) and therefore did not discriminate the groups based on gender. Thus, the combination of drive for thinness, body dissatisfaction, binge eating, emotion recognition, and emotion regulation significantly distinguished men and women, whereas measures of alexithymia (identifying and describing feelings) and social phobia did not.

**TABLE 3:** Standardized discriminant function coefficients, structure coefficients, and squared structured coefficients for each variable for the synthetic function and the centroids for men and women to explain the main effect of gender.

Variable	Standardized *r*_*s*_	Raw *r*_*s*_	*r*_*s*_^2^
**EDI-DT**	**0.62**	**0.76**	**0.58**
**EDI-BD**	**0.59**	**0.74**	**0.55**
**DERS**	**−0.29**	**−0.13**	**0.02**
**RMET**	**0.29**	**0.32**	**0.10**
**EDI-B**	**−0.26**	**0.17**	**0.03**
TAS-DIF	−0.14	−0.13	0.02
SPIN	−0.09	0.03	0.00
TAS-DDF	0.08	−0.07	0.01

	**Centroids**	**95% CI**	
		**Lower**	**Upper**

Men	−0.84	−0.97	−0.69
Women	0.49	0.63	0.83

**EDI-DT, Eating Disorder Inventory (EDI) Drive for Thinness subscale; EDI-BD, EDI Body Dissatisfaction subscale; DERS, Difficulties in Emotion Regulation Scale; RMET, Reading the Mind in the Eyes Task; EDI-B, EDI Bulimia subscale; TAS-DIF, Toronto Alexithymia Scale (TAS) Difficulties Identifying Feelings subscale; SPIN, Social Phobia Inventory; TAS-DDF, TAS Difficulty Defining Feelings subscale; 95% CI, 95% Confidence Interval of Centroid; r_*s*_, structure coefficient; r_*s*_^2^, squared structure coefficients; Bold typeface = standardized structure coefficient > 0.17. negative centroids indicate sign reversal of structure coefficients for that group. Variables are ordered by the absolute value of the standardized r_*s*_.**

Examination of the group centroids indicates that women were positively defined by this function, whereas men were negatively defined by this function. Women were defined by higher scores than men on EDI-DT, EDI-BD, and RMET. Men were defined by high scores on the DERS and EDI-B. The two groups’ centroids significantly differed, Welch’s *t*(263.83) = 10.65, *p* < 0.001, *d* = 1.27.

### Collapsing Athlete Categories

As our main hypotheses about team type were not supported in this sample, we conducted a *post hoc* analysis comparing all athletes (*n* = 183) to non-athletes (*n* = 96). The distribution of women was similar in athletes [*n* = 108 (60%)] and non-athletes [*n* = 68 (70%)], χ^2^(1) = 3.78, *p* = 0.052 *V* = 0.12.

**FIGURE 1:**
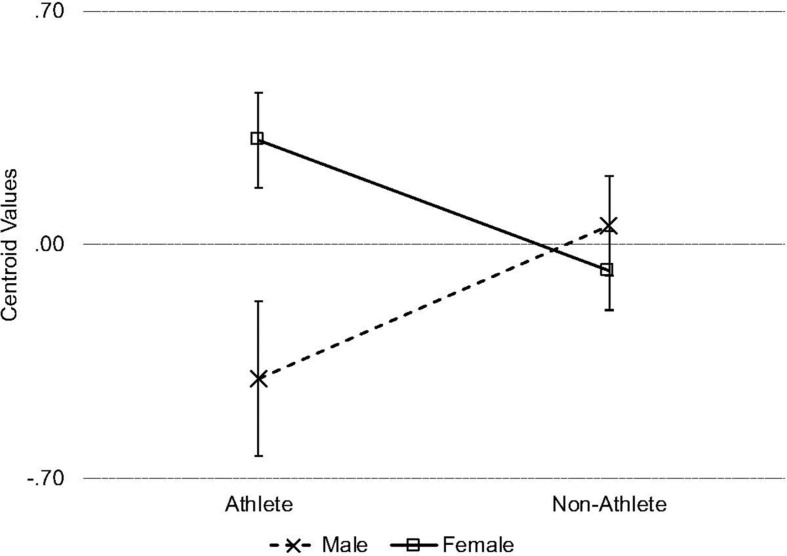
Line chart depicting the centroid values for the interaction effect of gender and athletics on the discriminant function. Error bars represent 1 SEM.

Again, results of the omnibus MANOVA showed a significant main effect of gender, *F*(8, 268) = 9.89, *p* < 0.001, η*_*p*_*^2^ = 0.228. The main effect of athletics was not significant, *F*(8, 268) = 1.06, *p* = 0.389, η*_*p*_*^2^ = 0.031. However, the gender × athletics interaction was significant *F*(8, 268) = 2.16, *p* = 0.031, η*_*p*_*^2^ = 0.061. The results of the DDA for the main effect of gender are identical to the analyses in the previous section. We proceeded to conduct a DDA on the gender × athletics interaction following the guidelines of Barton et al. (2016). The interaction accounted for approximately 6% of the variance, Wilks Λ = 0.942, *p* = 0.039, *R*_*c*_^2^ = 0.058. Results of the discriminant function are presented in Table 4, and the direction of the interaction is presented in Figure 1. As shown by the standardized canonical coefficients, the function was defined positively by the EDI-DT, DERS, and EDI-B scores. It was also negatively defined by TAS-DIF, and EDI-BD. TAS-DDF, SPIN, and RMET were not meaningful contributors to the composite function (standardized *r*_*s*_ < 0.17). Thus, the combination of drive for thinness, body dissatisfaction, binge eating, emotion dysregulation, and difficulty identifying feelings contributed to group differences between male and female athletes and non-athletes, whereas social phobia and emotion recognition did not.

**TABLE 4:** Standardized discriminant function coefficients, structure coefficients, and squared structured coefficients for each variable in the interaction gender and athletics.

Variable	Standardized *r*_*s*_	Raw *r*_*s*_	*r*_*s*_^2^
**TAS-DIF**	**−0.92**	**−0.92**	**0.85**
**EDI-DT**	**0.84**	**0.94**	**0.89**
**DERS**	**0.74**	**0.75**	**0.56**
**EDI-BD**	**−0.38**	**−0.42**	**0.17**
**EDI-B**	**0.30**	**0.30**	**0.09**
**TAS-DDF**	**0.20**	**0.20**	**0.04**
SPIN	−0.16	−0.16	0.03
RMET	−0.08	−0.09	0.01

	**Centroids**	**95% CI**	
**Athlete**		**Lower**	**Upper**

Men	−0.40	−0.68	−0.12
Women	0.31	0.08	0.55
**Non-athlete**			
Men	0.06	−0.37	0.22
Women	−0.08	−0.40	0.51

**TAS-DIF, Toronto Alexithymia Scale (TAS) Difficulties Identifying Feelings subscale; EDI-DT, Eating Disorder Inventory (EDI) Drive for Thinness subscale; DERS, Difficulties in Emotion Regulation Scale; EDI-BD, EDI Body Dissatisfaction subscale; EDI-B, EDI Bulimia subscale; TAS-DDF, TAS Difficulty Defining Feelings subscale; SPIN, Social Phobia Inventory; RMET, Reading the Mind in the Eyes Task; r_*s*_, structure coefficient; r_*s*_^2^, squared structure coefficients; 95% CI, 95% Confidence Interval of Centroid; Bold typeface = standardized structure coefficient > 0.17. Negative centroids indicate sign reversal of structure coefficients for that group. Variables are ordered by the absolute value of the standardized r_*s*_.**

Examination of the centroid value showed that male athletes were negatively defined by this function, whereas female athletes were positively defined by this function. Male and female non-athletes were not well defined by this function. The difference in definition between male and female athletes was significant (*p* < 0.001, *d* = 0.63). Female athletes were more positively defined by this function than female non-athletes (*p* = 0.041, *d* = 0.30). Male athletes did not significantly differ in their definition by this function from male non-athletes (*p* = 0.093, *d* = 0.40) and female non-athletes (*p* = 0.100, *d* = 0.28). Male and female non-athletes did not significantly differ from each other in their definition by this function (*p* = 0.623, *d* = 0.10) Thus, female athletes, compared to male athletes and female non-athletes, were defined by higher scores on the EDI-DT, DERS, and EDI-B. Male athletes, compared to female athletes and male non-athletes, were defined by greater scores on the TAS-DIF and EDI-BD.

## Discussion

The present study sought to address a substantial gap in the literature comparing psychosocial risk factors for eating disorders between male and female undergraduate athletes (Papathomas and Lavallee, 2012; Papathomas and Petrie, 2014; De Bruin, 2017). Few studies have probed the role of teammate interdependence (i.e., whether teammates are involved in the competitive aspect of a sport) on eating disorder risk (Evans et al., 2012; Eys et al., 2015). In the present study, we examined the relation of disordered eating to components of emotion regulation, social cognition, gender, and teammate interdependence (i.e., non-athletes, team athletes, and independent athletes). We hypothesized that difficulties in social cognition would correspond to a greater risk of disordered eating for female team athletes, compared to male team athletes and non-athletes. This hypothesis was not supported. Further, we did not observe a significant influence of teammate interdependence on emotional and social processes related to eating disorder risk. However, two main findings emerged in the present data set that are potentially important to better understand gender differences in eating disorder risk in undergraduates and undergraduate athletes. First, the psychosocial variables corresponded to a latent variable that significantly differentiated men and women in the present sample. Importantly, men and women differed in their presentation of risk factors and not in terms of severity. Were men and women simply to differ in severity on the latent factor, both centroids would have been in a similar direction with one much larger than the other. Second, our hypotheses regarding teammate interdependence were not supported, however when we combined individual and team athletes into one category, a significant interaction of gender and athletics emerged. The results showed that both male and female athletes were better defined by the latent function than male and female non-athletes. The variables that convey eating disorder risk differed in their presentation between male and female athletes: male athletes were defined by high scores on the EDI-BD and TAS-DIF, whereas female athletes were defined by high scores on the DERS, EDI-B, and TAS-DDF. In other words, female athletes had a similar presentation to disordered eating risk as men did in the main effect of gender (and *vice versa*). To our knowledge, this is the first study to establish these gender differences in undergraduate athletes who were predominantly at the club and recreational level.

There are several possible explanations for why we did not observe the hypothesized effects of teammate interdependence. It could be that the effects are more subtle than we had anticipated, and as such, the sample was underpowered to detect them. Although body dissatisfaction and disordered eating are frequently observed in highly competitive athletes (e.g., collegiate, professional, elite) (Kerr-Gaffney et al., 2018; Palermo and Rancourt, 2019; Scott et al., 2019a), our sample was predominantly (64%) engaged in club and recreational levels of athletics. It may be that differences in eating disorder risk as a function of teammate interdependence are better observed in more competitive environments. The athlete triad, for example, has been observed more frequently in elite sports than lower-level sports (Torstveit and Sundgot-Borgen, 2005; Torstveit et al., 2008; Sundgot-Borgen and Torstveit, 2010). Higher-level athletes may experience greater psychosocial risk factors associated with body dissatisfaction, such as pressure from coaches and teammates to lose or maintain weight or gain muscle mass (Baum, 2006; Holm-Denoma et al., 2009; De Bruin, 2017). In addition to structural level of competition, future work should probe self-rated importance and competitiveness in athletics, which has been identified as a better predictor of eating disorder risk than division level (Perelman et al., 2018; Palermo and Rancourt, 2019). It is also possible the hypothesized effects may be limited to youth as opposed to young adults; previous work analyzing the influence of teammate interdependence found effects in samples of children and adolescents (Haase, 2009; Frisen and Holmqvist, 2010; Morano et al., 2011; Dyremyhr et al., 2014). Finally, it could be that the mix of sport types (e.g., lean; non-lean; gravitational; aesthetic) diluted the anticipated effects. The athlete triad (for men and women) has been observed most in sports where weight management is either required (e.g., wrestling) or provides an advantage, such as in endurance or aesthetic sports (e.g., gymnastics) (Sundgot-Borgen and Torstveit, 2010; De Souza et al., 2019). Nevertheless, interesting and potentially important findings emerged pertaining to gender and athletics more broadly.

### Main Effects of Gender

There was a main effect of gender in both the team type × gender and athletics × gender models. Two key patterns emerged within the discriminant function as a function of gender. Compared to men, women were defined by higher scores on the EDI-DT (drive for thinness) and EDI-BD (body dissatisfaction). Women being defined by higher scores than men on the EDI-DT and EDI-BD is not surprising. These subscales have been criticized as biased toward feminized body dissatisfaction and the thin ideal, and therefore, they may not adequately capture the body shape concerns that boys and men typically experience (Stanford and Lemberg, 2012; Elosua and Hermosilla, 2013; Pope et al., 2015; Chapman and Woodman, 2016). Namely, boys and men tend to pursue larger (e.g., more muscular) bodies, whereas girls and women tend to pursue a thinner body type (Elosua and Hermosilla, 2013; Japil et al., 2017; Fernandez-Bustos et al., 2019). Additionally, the questions on the EDI-DT and EDI-BD focus on satisfaction with the shape of parts of the body for which women, and not men, frequently experience dissatisfaction (e.g., hips and buttocks). Men often focus on abdominals, arms, and chest muscles (Crossley et al., 2012; Cordes et al., 2016). It is possible that “body dissatisfaction” in male participants may have been better captured in a different instrument (Stanford and Lemberg, 2012; Smith et al., 2017).

Compared to women, men were defined by higher scores on the EDI-B and lower scores on the RMET and the DERS. Whereas the EDI-DT and EDI-BD focus on cognitive aspects of eating disorder risk (i.e., worry and dissatisfaction with one’s body), the EDI-B subscale measures behavioral aspects of binge eating (e.g., “I stuff myself with food”) (Stanford and Lemberg, 2012). These results suggest that difficulties in emotion recognition, emotion regulation, and binge-eating correspond to distinguish men from women. Although men tend to perform worse on the RMET than women (Kirkland et al., 2013), to our knowledge, this is the first study to identify performance on the measure as contributing to gender differences in eating behavior. Just two previous studies examined RMET scores and eating disorder risk in men, and both reported null findings (Goddard et al., 2014; Griffiths et al., 2014). Goddard et al. (2014) and Griffiths et al. (2014) assessed eating restraint, whereas we found an association with binge eating. Therefore, it is possible that the RMET better measures emotional processes (e.g., theory of mind, emotion recognition) associated with risk of binge eating than restriction.

Gender differences in the DERS are infrequently reported (e.g., Ritschel et al., 2015; Hallion et al., 2018). Univariate analyses in the present sample confirmed that men and women did not significantly differ (*p*’s > 0.05) on any subscale or the sum score of the DERS (data not shown). Our findings are consistent with previous work that identified gender differences in the downstream consequences of emotion dysregulation, but not levels of emotion dysregulation itself (Kret and De Gelder, 2012; Di Tella et al., 2020). Further, these results suggest that emotion recognition of others – assessed via the RMET – also corresponds to emotion dysregulation and binge eating. Taken together, the present results reflect previous findings suggesting that men, more so than women, use binge eating as a maladaptive coping mechanism for emotional distress (Han and Pistole, 2014; Kukk and Akkermann, 2017; Racine et al., 2019).

### Interaction of Athletics and Gender

Several notable findings emerged when we simply compared athletes to non-athletes. Male and female athletes were well-defined by the discriminant function, yet male and female non-athletes were not. Women were defined by greater EDI-DT regardless of athlete status (Elosua and Hermosilla, 2013; Pope et al., 2015; Chapman and Woodman, 2016). Male athletes, compared to female athletes, were defined by greater scores on the EDI-BD and TAS-DIF. For male athletes, the increased scores on the EDI-BD may correspond to increased desire for muscularity in Labre (2005), Galli and Reel (2009), Olson et al. (2009), Crossley et al. (2012) and Cordes et al. (2016). Thus, the male athlete’s heightened EDI-BD may not reflect a desire to lose weight or reduce adiposity, but rather a dissatisfaction with muscle mass and other aspects of physical appearance.

To our knowledge, this is the first study to observe a stronger association between difficulty identifying feelings and body dissatisfaction for male athletes than female athletes. Only one previous study has examined gender differences in the association between alexithymia and disordered eating, and the results showed a stronger association between TAS-DIF and disordered eating risk for women than men (Iacolino et al., 2017). Therefore, it is difficult to compare this finding to previous research. Although women report more sensitivity to bodily signals, men are more likely to classify bodily signals as emotional distress (Grabauskaite et al., 2017). It may be that this focus on the body as a source of emotional distress transitions into concern about body shape (in this case, muscularity) more so for male athletes than female athletes (Núñez-Navarro et al., 2012; Dakanalis and Riva, 2013; Wyssen et al., 2016).

Compared to male athletes, female athletes were better defined by higher scores on the DERS and EDI-B. Men exhibited a similar pattern (with lower scores on the EDI-BD) in the main effect of gender. Female athletes were also partially defined by higher scores on the TAS-DDF, which, together with greater DERS and EDI-B, reflects previous work that found emotion dysregulation and alexithymia contribute to binge eating risk for female athletes (Wollenberg et al., 2015; Shriver et al., 2016). It may be that female athletes engage in binge eating as a means of emotion regulation, similar to men in the general population (Han and Pistole, 2014; Kukk and Akkermann, 2017; Racine et al., 2019). Female athletes also report higher perceived stress than male athletes and may binge eat as a coping mechanism (Williams, 2016). Relatedly, female athletes may binge eat as a reaction to restrictive eating, which is more common in female athletes than male athletes (Engel et al., 2003; Fay et al., 2011). Importantly, the cross-sectional nature of this study precludes a conclusion about causality. Longitudinal studies are needed to clarify this relation. Given a comparatively higher focus on restrictive eating in athletes, more work is needed to confirm and better understand why female athletes may engage in binge eating.

The RMET contributed to the main effect of gender, but not the gender × athletics interaction. A recent meta- analysis examining gender differences in the RMET identified a significant, consistent pattern of superior performance by women compared to men, although the effect size was small (*g* = 0.114) (Kirkland et al., 2013). It may be that this effect is too small to detect when the present sample was stratified into athletes and non-athletes. Given how the RMET contributed to the main effect of gender, future researchers should consider including this assessment as a behavioral measure of recognizing emotional expression in others that may measure different components of emotion regulation than self-report.

It is worth noting that the SPIN did not significantly contribute to either the discriminant function for the effect gender or the discriminant function for the gender × athletics interaction. The SPIN was positively correlated with each dependent variable for both men and women (Table 2). When a variable is correlated across several variables, its explanatory power in DDA is reduced (Sherry, 2006; Barton et al., 2016). This result is consistent with previous work that found social phobia to be associated with components of emotional processing, disordered eating, and body dissatisfaction in men and women (Keel and Forney, 2013; Kerr-Gaffney et al., 2018; Barnes et al., 2020). Previous researchers found elevated symptoms of social phobia to be so common in undergraduates that responses on social phobia inventories do not distinguish respondents with and without diagnosis (Stewart and Mandrusiak, 2007). Taken together, it may be that the SPIN does not capture components of social phobia that distinguish subgroups of undergraduates.

## Limitations

The present study identified some important distinctions between men and women, particularly as related to athletics. However, there are some important limitations that should be noted. The present study was cross-sectional, and data were self-reported from a self-selected and predominantly Caucasian sample at one undergraduate university, which limits conclusions about causality and generalizability. Future researchers would do well to collect data from multiple campuses or execute longitudinal studies to address this limitation. The EDI-3 was developed primarily with women, which may not capture eating behavior and body shape concerns of athletes of all genders (Stanford and Lemberg, 2012; Chapman and Woodman, 2016). Although many of the included instruments used in the present study have been used amply within collegiate athlete samples, only the EDI has been independently validated for this population (Pope et al., 2015). Future research should establish measurement equivalence/invariance for these measures in an athletic sample. It is also important to note that we did not inquire or determine eating disorder diagnosis as we were interested in disordered eating risk more generally. Finally, because we were interested in the influence of team dynamics, our classification of non-athletes included those who engaged in personal exercise but not organized exercise, which may also convey similar eating disorder risk to those involved in organized sports (Bratland-Sanda et al., 2015; Fernandez-Bustos et al., 2019). Given the observed differences between non-athletes and athletes, we do not believe that engagement in personal fitness influenced the results of this study. Future work would do well to monitor this potentially informative variable.

## Conclusion

This study contributes to the limited research that has compared young men and women on psychosocial risk of disordered eating. Although we did not find differences in eating disorder risk across team, independent, or non-athletes, important findings emerged that suggest male and female athletes, more broadly, experience different types of body dissatisfaction and likely pursue different body ideals. Crucially, most athletes in this study were recreational and club athletes, indicating that elite athlete status is neither necessary nor sufficient to convey risk of eating disorders. These results suggest that many of the psychosocial and socioemotional risk factors for disordered eating in the general population are also present in the undergraduate athlete population. This study adds to the growing research that has found risk factors between men and women differ in terms of presentation, but not necessarily in severity (Perelman et al., 2018). Programs designed to prevent and treat disordered eating in athlete populations should tailor these approaches to the different needs of men and women. More work is needed to better understand the psychosocial risk factors for disordered eating in athletes.

## Data Availability Statement

The datasets generated for this study are available on request to the corresponding author.

## Ethics Statement

The studies involving human participants were reviewed and approved by the Institutional Review Board, Towson University. The patients/participants provided their written informed consent to participate in this study.

## Author Contributions

RW and CT conceived, designed, and conducted the study. EB conducted statistical analysis. RW, EB, and CT drafted the manuscript. All authors contributed to the article and approved the submitted version.

## Conflict of Interest

The authors declare that the research was conducted in the absence of any commercial or financial relationships that could be construed as a potential conflict of interest.

## Footnotes

1 As defined by the Student Life office of the university from which the sample was recruited: *recreational* sports (i.e., intramural sports) take place on-campus and against other students from within the university; *club* sports are student-run organizations that compete against other universities both on- and off-campus.

2 There were six multivariate outliers based on Mahalanobis (*D*^2^) distances (*p*’s < 0.001). Removing these individuals did not alter significance or pattern of the results below.
