# Supplementary material for: Difficulties in Emotion Regulation, Alexithymia, and Social Phobia Are Associated With Disordered Eating in Male and Female Undergraduate Athletes
Source: Front Psychol. 2020 Jul 22;11:1646. doi: 10.3389/fpsyg.2020.01646 (PMC7387713; doi:10.3389/fpsyg.2020.01646)
Supplement: Supplementary file 1 [file Data_Sheet_1.docx]

| Appendix A: Frequency of primary sport selected | | | | |
| --- | --- | --- | --- | --- |
| Sport | Individual or Team | *n* Men | *n* Women | Total |
| Baseball | Team | 9 | 0 | 9 |
| Basketball | Team | 8 | 9 | 17 |
| Boxing^*^ | Individual | 2 | 0 | 2 |
| Cheerleading | Team | 0 | 4 | 4 |
| Dance | Team | 0 | 11 | 11 |
| Field Hockey | Team | 0 | 16 | 16 |
| Figure Skating | Individual | 0 | 2 | 2 |
| Flag football | Team | 1 | 1 | 2 |
| Football | Team | 6 | 0 | 6 |
| Golf | Individual | 2 | 0 | 2 |
| Gymnastics | Individual | 0 | 2 | 2 |
| Hockey | Team | 5 | 0 | 5 |
| Lacrosse | Team | 10 | 3 | 13 |
| Marching Band | Team | 0 | 1 | 1 |
| Martial Arts | Individual | 1 | 1 | 2 |
| Paintball | Individual | 1 | 0 | 1 |
| Rock Climbing^*^ | Individual | 1 | 0 | 1 |
| Rugby | Team | 4 | 1 | 5 |
| Running | Individual | 0 | 12 | 13 |
| Soccer | Team | 16 | 13 | 29 |
| Softball | Team | 0 | 3 | 3 |
| Surfing^*^ | Individual | 0 | 1 | 1 |
| Swimming | Individual | 2 | 7 | 9 |
| Tennis | Individual | 1 | 7 | 8 |
| Ultimate Frisbee | Team | 2 | 2 | 4 |
| Volleyball | Team | 1 | 13 | 14 |
| Wrestling | Individual | 2 | 0 | 2 |
| *Non-Athlete* |  | *28* | *68* | *96* |
| **Whole Sample** |  | **102** | **177** | **279** |
| *Notes*: The following responses were combined: Running, Cross Country, and Track; Basketball and Women’s Basketball; “Pom Squad” and Dance; Karate, Mixed Martial Arts; mentions of “club” or “pro” of a sport were also combined.  ^*^Under the classification of Evans et al. (2012), boxing, surfing, and rock climbing may be considered “solitary” rather than “individual,” but for the purposes of this study, they are coded as “individual;” moreover, the University from which the sample was drawn had organized competitive clubs for these sports. | | | | |
